# Thiol Based Redox Signaling in Plant Nucleus

**DOI:** 10.3389/fpls.2018.00705

**Published:** 2018-05-28

**Authors:** Laura Martins, José Abraham Trujillo-Hernandez, Jean-Philippe Reichheld

**Affiliations:** ^1^Laboratoire Génome et Développement des Plantes, Université Perpignan Via Domitia, Perpignan, France; ^2^Laboratoire Génome et Développement des Plantes, Centre National de la Recherche Scientifique, Perpignan, France

**Keywords:** nucleus, thiol, glutathione, thioredoxin, glutaredoxin, ROS

## Abstract

Reactive oxygen species (ROS) are well-described by-products of cellular metabolic activities, acting as signaling molecules and regulating the redox state of proteins. Solvent exposed thiol residues like cysteines are particularly sensitive to oxidation and their redox state affects structural and biochemical capacities of many proteins. While thiol redox regulation has been largely studied in several cell compartments like in the plant chloroplast, little is known about redox sensitive proteins in the nucleus. Recent works have revealed that proteins with oxidizable thiols are important for the regulation of many nuclear functions, including gene expression, transcription, epigenetics, and chromatin remodeling. Moreover, thiol reducing molecules like glutathione and specific isoforms of thiols reductases, thioredoxins and glutaredoxins were found in different nuclear subcompartments, further supporting that thiol-dependent systems are active in the nucleus. This mini-review aims to discuss recent progress in plant thiol redox field, taking examples of redox regulated nuclear proteins and focusing on major thiol redox systems acting in the nucleus.

## Introduction

Oxygen is one of the most important molecules for aerobic organisms. It is necessary for cell metabolism, but it also generates reactive oxygen species (ROS) as by-products of oxidoreduction pathways. ROS include free radical species like superoxides ($O_{2}^{•-}$), hydroxyl radicals (OH^•^), or nitric oxide (NO^•^), and non-radical species like hydrogen peroxide (H_2_O_2_) and peroxynitrite (ONOO^-^) (Sies et al., 2017). In plants, major sources of ROS are photosynthetic and respiratory chains in chloroplasts and mitochondria. ROS are also generated by plasma membrane NADPH oxidases and peroxisomal xanthine oxidases. Oxidative eustress is playing important signaling functions by inducing post-translational modifications (PTM) and by regulating protein redox state. ROS can also trigger oxidative distress which can damage the cell (Foyer and Noctor, 2016; Choudhury et al., 2017). Plant cells display a large panel of ROS scavenging enzymes like catalases, peroxidases, and superoxide dismutases. They also generate compounds that reverse ROS-induced oxidations. Among these compounds are antioxidant molecules like glutathione and ascorbate, which both play important roles as cofactors for thiol reduction enzymes like peroxidases and reductases (Noctor, 2017; Rahantaniaina et al., 2017). Glutathione and ascorbate are themselves reduced by glutathione reductases (GRs) and dehydroascorbate reductases (DHARs). Thioredoxins (TRXs) and glutaredoxins (GRXs) are key thiol reduction enzymes. They act as reducing power of metabolic enzymes and ROS scavenging systems but they also regulate thiol-based post-transcriptional redox modifications in proteins (Meyer et al., 2012). Oxidized TRXs are generally reduced by NADPH-dependent thioredoxin reductases (NTRs), whereas the reduction of GRXs is dependent on glutathione. Due to their multifunctional thiol reduction capacities, TRXs and GRXs have been involved in many metabolic functions, controlling plant developmental programs and acting as key signaling molecules in response to abiotic and biotic stresses (Meyer et al., 2009; Rouhier et al., 2015). In this mini-review, we aim to give an updated overview of nuclear thiol-based ROS signaling in plants.

## Ros and Cys Ox-PTMs in the Nucleus

Some data suggest that ROS are actively generated in the nucleus (Ashtamker et al., 2007), but they principally accumulate in the nucleus through transfer from other cell compartments. Genetically encoded fluorescent H_2_O_2_ sensors (e.g., HyPer) have consistently shown that cytosolic H_2_O_2_ freely diffuses in the nucleus through nuclear pores (Møller et al., 2007; Rodrigues et al., 2017). It is also transferred from the chloroplasts to the nucleus under pathogen and high light (HL) conditions (Caplan et al., 2015; Exposito-Rodriguez et al., 2017).

**FIGURE 1:**
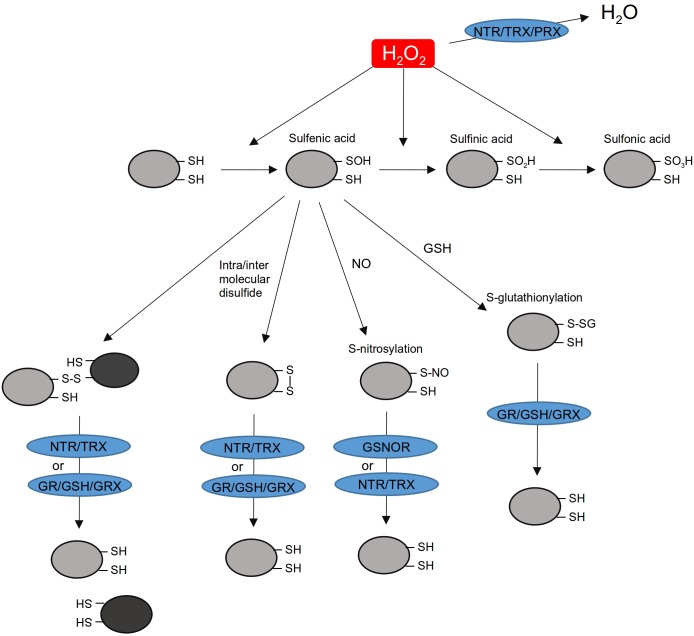
H_2_O_2_-induced thiol modifications and scavenging activities in the nucleus. H_2_O_2_ accumulating in the nucleus can be detoxified by a NTR/TRX/PRX system. While many other H_2_O_2_ detoxification enzymes are active in plants (e.g., catalases and ascorbate peroxidases), their presence in the nucleus is not demonstrated yet. They are not represented here. H_2_O_2_ can oxidize thiol residues in protein. Sulfenic acid reacts with GSH, NO or with adjacent thiol residues. Putative nuclear proteins prone to S-glutathionylated, S-nitrosylated, or disulfide bonds formation have been identified by proteomic and biochemical approaches (see Supplementary Table 1; Delorme-Hinoux et al., 2016; Pérez-Pérez et al., 2017). S-glutathionylated, S-nitrosylated, or intra/intermolecular disulfide bonds can be reduced by NTR/TRX, GR/GSH/GRX, or GSNOR. NTR, NADPH-dependent thioredoxin reductase; TRX, thioredoxin; GR, glutathione reductase; GRX, glutaredoxin; PRX, peroxiredoxin; GSNOR, S-nitrosoglutathione reductase; GSH, glutathione; NO, nitric oxide; H_2_O_2_, hydrogen peroxide.

The chemical characteristics of the sulfur atom make Cys and Met residues major sites of oxidation within proteins (Davies, 2005). Depending on their pKa and on the pH of the medium, thiol residues are deprotonated into a thiolate residue (R-S^-^) which is prone to oxidation. This is leading to successive oxidations to sulfenic (R-SOH), sulfinic (R-SO_2_H), and sulfonic (R-SO_3_H) acids (Davies, 2005). Thiol groups can also form a disulfide bridge (S-S) or react with reactive nitrogen species (RNS) or oxidized glutathione (GSSG) resulting in S-nitrosylation (R-SNO) or S-glutathionylation (R-S-SG). Depending on their nature, most of these thiol modifications can be reversed by dedicated thiol reduction systems (TRX, GRX, and GSNO Reductase) which exhibit disulfide bond, deglutathionylation or denitrosylation activities (**Figure 1**). Thiol modifications can alter the structure and/or the activity of many proteins like transcription factors, MAP kinases, and chromatin modification proteins (see below). Proteomic approaches aiming to identify oxidized thiol targets have been developed in plants. Hundreds of nuclear candidate proteins were found sulfenylated, nitrosylated, or glutathionylated (Supplementary Table 1; Zaffagnini et al., 2012, 2016; Morisse et al., 2014; Waszczak et al., 2014; Chaki et al., 2015; Pérez-Pérez et al., 2017). These approaches also revealed the complexity of the thiol redox modification networks in plants (Pérez-Pérez et al., 2017). However, among all these candidates, redox regulation has been validated only in a few cases (see below).

## Thiol Redox Systems in the Nucleus

### Glutathione

Plants exhibit a large panel of thiol reduction systems (Meyer et al., 2012). Among them is glutathione, a low molecular weight thiol-containing tripeptide (γ-glutamyl-cysteinyl-glycine). Glutathione biosynthesis is performed in chloroplasts and in the cytosol but is found in almost all cell compartments, including the nucleus. Nuclear pores are generally assumed to allow unrestricted bidirectional diffusion of glutathione across the nuclear envelope. Therefore, nuclear glutathione translocation to the nucleus can be passive. Glutathione quantification at the subcellular level is technically challenging due to its highly dynamic compartmentation (Delorme-Hinoux et al., 2016). Thiol-specific dyes and genetically encoded probes such as reduction-oxidation-sensitive green fluorescent proteins (roGFPs) have consistently detected glutathione in the nucleus. Immunocytochemistry (ICC) coupled to electronic microscopy were also used to address its location at a subcompartment level (Zechmann et al., 2008; Zechmann and Müller, 2010). This study found glutathione uniformly spread in the nucleoplasm, without distinction between euchromatin and heterochromatin. In *Arabidopsis thaliana*, a glutathione reductase (GR1) is found in the nucleus, suggesting that oxidized glutathione (GSSG) is actively reduced in the nucleus (Delorme-Hinoux et al., 2016).

The functions of glutathione in the nucleus are still poorly understood. Thiol-labeling experiments using the 5-chloromethylfluorescein diacetate (CMFDA) dye and glutathione redox state measured by roGFP, suggest that a redox cycle is occurring during the cell cycle progression and is critical for cell cycle progression (Diaz Vivancos et al., 2010; Vivancos et al., 2010; de Simone et al., 2017). Consistently, sustained mild oxidation observed in ascorbate mutants also restricts nuclear functions and impairs progression through the cell cycle (de Simone et al., 2017). More than acting as a general redox buffer, glutathione could also provide reducing moiety for anti-oxidant enzymes like GRXs (i.e., GRXC1, GRXC2, GRXS17, GRXS13, and ROXY1/2/4) and Glutathione S-Transferases (i.e., GSTF5-10, GSTU19/20, and GSTT19/20), several of them having been identified in the nucleus (Rouhier et al., 2015; Palm et al., 2016).

Consistent levels of ascorbate have also been found in the nucleus but little evidence for its nuclear functions has been described (Zechmann et al., 2011; Considine and Foyer, 2014; de Simone et al., 2017; Zechmann, 2017).

### Thiols Reductases in the Nucleus

**FIGURE 2:**
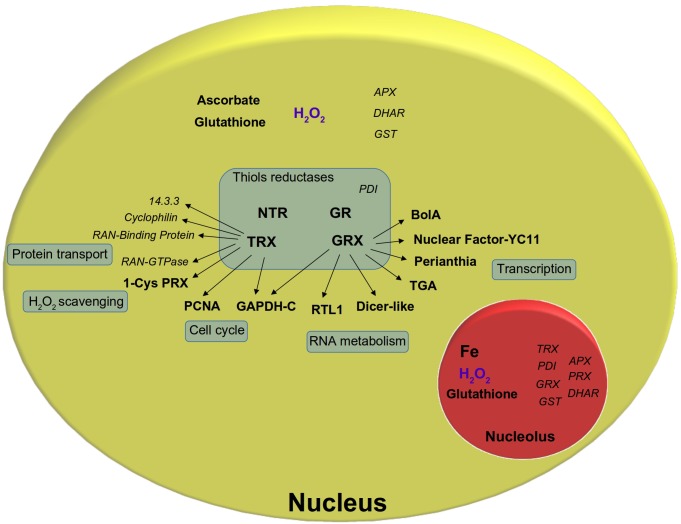
Major thiol redox components found in the nucleus. Thiol reduction systems, thiol-containing target proteins were represented as well as ROS scavenging enzymes. Proteins which were only found in proteomic data (according to Montrichard et al., 2009; Waszczak et al., 2014; Delorme-Hinoux et al., 2016; Palm et al., 2016; Montacié et al., 2017; Pérez-Pérez et al., 2017) but not further validated in the nucleus were represented *in italic*. NTR, NADPH-dependent thioredoxin reductase; TRX, thioredoxin; GR, glutathione reductase; GRX, glutaredoxin; PRX, peroxiredoxin; PDI, protein disulfide isomerase; GST, glutathione S-transferase; APX, ascorbate peroxidase; DHAR, dehydroascorbate reductase; Ran-GTPase, Ran-guanosine triphosphatase; PCNA, proliferating cell nuclear antigen; GAPDH-C, glyceraldehyde 3-phosphate dehydrogenase-C; RTL1, RNAse III-like 1; TGA, TGA-transcription factor; NF-YC11, nuclear factor-YC11; GSH, glutathione; NO, nitric oxide; H_2_O_2_, hydrogen peroxide.

Thioredoxins (TRXs) and Glutaredoxins (GRXs) are major classes of thiols reductases. Plants harbor a complex TRX and GRX network (Meyer et al., 2012). Among the 40 TRXs and 50 GRXs isoforms were found in *Arabidopsis*, at least 4–8 of them have been assigned to the nucleus, although often in association with a cytosolic localization (Delorme-Hinoux et al., 2016). Moreover, NTR isoforms were also found in the nucleus, where they reduce TRXh, TRXo1, and Nucleoredoxin 1 (NRX1) (Serrato et al., 2001; Serrato and Cejudo, 2003; Marty et al., 2009; Marchal et al., 2014). Some thiol reductases are constitutively located in the nucleus, but others were found to shuttle between the cytosol and nucleus. In tomato subjected to heat stress, the predominant cytosolic GRXS17 was found to relocate in the nucleus (Wu et al., 2012). In wheat and Chlamydomonas, TRXh isoforms accumulate in the nucleus upon oxidative or genotoxic stress **(**Serrato and Cejudo, 2003; Sarkar et al., 2005). Little is known about the subnuclear localization of these respective proteins. This is due to the low resolution of localization techniques like ICC and GFP-fusion coupled with confocal microscopy analyses. In most cases, these proteins are detected in the nucleoplasm, without distinction between heterochromatin and euchromatin, and are apparently excluded from the nucleolus. Recently, ICC analyses have detected NRX1 and NTRA in the nucleolar cavity, but the functional significance of this localization is still unknown (Marchal et al., 2014). Major thiol redox components found in the nucleus are presented in **Figure 2**. ROS scavenging enzymes and thiol-containing target proteins are shown as well. Most of these components have been recently reviewed by Delorme-Hinoux et al. (2016) and will not be further discussed here.

The nucleolus, a nuclear subcompartment responsible for rRNA biosynthesis, might also be subjected to redox regulation (Saez-Vasquez and Medina, 2009). Significant accumulation of H_2_O_2_ has been detected in the nucleolus in tobacco cell suspension subjected to elicitor treatments (Ashtamker et al., 2007). Intriguingly, the nucleolus also accumulates high amounts of iron, which might provide substrates for ROS generation by Fenton reactions (Roschzttardtz et al., 2011). In addition, glutathione and several isoforms of PRXs, DHARs, APXs, TRXs, and GRX-like proteins were enriched in this compartment (Zechmann et al., 2008; Zechmann and Müller, 2010; Palm et al., 2016; Montacié et al., 2017). Whether redox activities are occurring in the nucleolus will need further investigations.

## Redox-Regulated Nuclear Functions

### Transcriptomic Control by ROS

Reactive oxygen species causes drastic changes in nuclear gene expression (Gadjev et al., 2006; Willems et al., 2016; Shaikhali and Wingsle, 2017). Oxidative stress affects many pathways involved in RNA processing, including splicing, polyadenylation, exporting, and editing. It is also involved in RNA degradation and protein translation (Van Ruyskensvelde et al., 2018). Under High-light (HL) conditions, ROS originated in chloroplasts are associated with chloroplast-to-nucleus (retrograde) signaling. Among the molecules involved in the retrograde signaling (Suzuki et al., 2012; Vogel et al., 2014; Dietz et al., 2016), singlet oxygen (^1^O_2_) induces expression of subsets of ^1^O_2_-responsive genes and enhances tolerance to HL and to other abiotic and biotic stress (Wagner et al., 2004; Carmody et al., 2016). H_2_O_2_ originating by dismutation of superoxide in the chloroplast has also been recently shown to be involved in retrograde signaling upon HL exposure (Exposito-Rodriguez et al., 2017). Another retrograde signaling was suggested to involve a redox regulation of the chloroplastic cyclophilin Cyp20.3, leading to stimulation of Cys synthesis, accumulation of non-protein thiols and activation of defense gene expression (Dominguez-Solis et al., 2008; Park et al., 2013). Presumably, ROS generated in other cell compartments (mitochondria, peroxisomes, and apoplast) can also exert similar retrograde signaling (Noctor and Foyer, 2016; Rodríguez-Serrano et al., 2016).

Photorespiration produces H_2_O_2_ in peroxisomes. In this compartment, catalases play an important role in removing H_2_O_2_. The *cat2* mutant inactivated in the major peroxisomal catalase accumulates a high level of peroxisomal H_2_O_2_ and impacts nuclear gene expression extensively, rapidly inducing subsets of stress and hormonal response genes (Queval et al., 2007). In this case, regulation of gene expression involves glutathione signaling also, as the transcriptomic response is partly abolished in a glutathione-defective (*cad2*) *cat2 cad2* double mutant (Han et al., 2013a,b). A signaling role of glutathione on nuclear gene expression was also suggested by transcriptomic data in genetically or pharmacologically manipulated glutathione backgrounds (Xiang and Oliver, 1998; Ball et al., 2004; Schnaubelt et al., 2015). Other transcriptomic analyses also showed involvement of thiol reduction systems in modulating nuclear gene expression (Bashandy et al., 2009; Martins et al., unpublished data). Whether these actors are directly involved in gene expression needs further investigations.

### Redox Regulation of Transcription Factors

A likely impact of ROS on nuclear gene expression relies on the regulation of redox-sensitive transcription factors (Considine and Foyer, 2014; Dietz, 2014; Rouhier et al., 2015; Waszczak et al., 2015). In most cases, redox regulation induces conformation changes in transcription factors or associated proteins. Such modifications can occur in the cytosol and trigger nuclear translocation, e.g., by uncovering of a nuclear localization sequence (NLS). A well-documented example is the thiol redox-dependent nuclear translocation of the glycolytic enzyme Glucose 6-Phosphate Dehydrogenase C (GAPDH-C) which impacts both its metabolic activity and its moonlighting function as a transcriptional activator of glycolytic genes (Holtgrefe et al., 2008; Vescovi et al., 2013; Zaffagnini et al., 2013; Testard et al., 2016; Zhang et al., 2017). The HL- and H_2_O_2_-dependent nuclear translocation of Heat-Shock Factors (HSFA1D and HSFA8) is also dependent on specific Cys residues (Miller and Mittler, 2006; Jung et al., 2013; Giesguth et al., 2015; Dickinson et al., 2018). The pathogen-induced Salicylic Acid (SA)-dependent transcriptional response is mediated by redox-dependent nuclear translocation of NON-EXPRESSOR OF PR GENES1 (NPR1). In this particular case, NPR1 is kept in the cytosol in a disulfide-bound oligomeric homocomplex. Upon pathogen attack, SA induces TRXh5 expression which counteracts NPR1 oligomer formation by reducing NPR1 disulfides. Moreover, through its denitrosylase activity, TRXh5 also suppresses the stimulatory effect of Cys156 S-nitrosylation on formation of disulfide-linked NPR1 oligomer (Tada et al., 2008; Kneeshaw et al., 2014). NPR1 is translocated in the nucleus where it promotes PR gene expression through interaction with TGA transcription factors such as TGA1 (Després et al., 2003; Mou et al., 2003; Tada et al., 2008; Kneeshaw et al., 2014).

Indeed, other members of the TGA transcription factors are likely redox regulated in the nucleus. Among the 10 TGA factors found in *Arabidopsis*, several of them (i.e., TGA1, TGA2, TGA3, TGA7, and Perianthia) interact with type III GRXs (ROXY1 and 2) and are involved in the development of petals, anthers and microspores. Although the redox dependent control of these TGA is not fully established, ROXY/TGA interactions are occurring in the nucleus and affect TGA-regulated gene expression (Xing et al., 2005; Xing and Zachgo, 2008; Li et al., 2009, 2011; Murmu et al., 2010; reviewed by Dietz, 2014 and Delorme-Hinoux et al., 2016).

R2R3-type MYB transcription factors from maize require reducing conditions for DNA binding. Under non-reducing conditions, Cys49 and Cys53 form a disulfide bond that prevents the R2R3 MYB domain from binding DNA (Williams and Grotewold, 1997; Heine et al., 2004). More recently, the structure and the DNA binding activity of a AtMYB30 transcription factor were shown to be influenced by S-nitrosylation (Tavares et al., 2014).

AP2/ethylene response factor (ERF) is another class of transcription factors which undergoes redox regulation (Welsch et al., 2007; Shaikhali et al., 2008; Vogel et al., 2014). One of the most striking examples was described for the Rap2.12-dependent regulation of hypoxia response genes. Under aerobic conditions, Rap2.12 is bound to the plasma membrane within an acyl-CoA binding protein 1 or 2 (ACBP1/2) complex. In low oxygen, Rap2.12 is released from the plasma membrane by a mechanism involving a N-terminal Cys2 residue, and is translocated to the nucleus where it activates hypoxia response genes (Gibbs et al., 2011; Licausi et al., 2011; Licausi, 2013).

Comelli and Gonzalez (2007) also reported a redox regulation of conserved Cys in the homeodomain (HD) DNA of plant class III HD-Zip proteins. Here, DNA binding capacities are only maintained when an intramolecular disulfide bond is reduced by a thioredoxin (Comelli and Gonzalez, 2007). A Cys-dependent redox regulation of the DNA binding activity of basic region leucine zipper (bZIP) transcription factors has also been reported (Shaikhali et al., 2012).

Finally, a subunit of the Nuclear Factor-Y (NF-Y) transcription factor complex (NY-YC11) physically interacts with the iron-sulfur cluster glutaredoxin GRXS17 in the nucleus. It is not known yet if this interaction is redox-dependent (Knuesting et al., 2015). In the cytosol and the nucleus, GRXS17 also interacts with and reduces BolA2, a factor involved in iron metabolism (Couturier et al., 2014; Qin et al., 2015).

### Epigenetic Regulation

Redox regulation of epigenetic processes has mostly been addressed in mammals (García-Giménez et al., 2017), but this field is poorly explored in plants (Delorme-Hinoux et al., 2016; Shen et al., 2016). Nevertheless, due to the ubiquity of these basic mechanisms in living organisms, it is likely that such regulation occurs in plants as well. Different enzymes involved in histone methylation are prone to redox regulation, affecting both positive and negative histone marks (e.g., H3K4me2, H3K4me3, H3K79me3, H3K27me2, and H3K9me2) (Chen et al., 2006; Zhou et al., 2008, 2010; Niu et al., 2015). In mammals, nuclear histone acetylation activities are redox sensitive, affecting chromatin conformation and transcription (Ito et al., 2004; Ago et al., 2008; Nott et al., 2008; Doyle and Fitzpatrick, 2010). During brain development, neurotrophic factors induce S-nitrosylation at conserved Cys of HDAC2 in neurons, resulting in changes of histone modification and gene expression (Nott et al., 2008). Upon cardiac hypertrophy, a ROS/TRX-dependent redox switch of key Cys residues affects nuclear trafficking of a class II HDAC and subsequent gene expression (Ago et al., 2008). Within the large family of HDAC identified in plants (Pandey et al., 2002), members of the class I RPD-3 like HDAC (HDAC9, 19) have been shown to be sensitive to oxidation (Liu et al., 2015; Mengel et al., 2017), but the physiological significance of those modifications is still poorly understood. NO-induced HDAC inhibition is proposed to operate in plant stress response by facilitating the stress-induced transcription of genes (Mengel et al., 2017).

In addition to methylation and acetylation, mammalian histone H3 has been shown to be glutathionylated on a conserved and unique Cys residue (García-Giménez et al., 2014). Histone H3 glutathionylation increases during cell proliferation and decreases during aging. This produces structural changes affecting nucleosome stability and leading to a more open chromatin structure (García-Giménez et al., 2013, 2014, 2017; Xu et al., 2014).

Small RNAs (siRNA and miRNA) are key regulators of gene expression, involved in most developmental and stress response processes in eukaryotic cells (Leisegang et al., 2017). Biogenesis of small RNAs is orchestrated by DICER-LIKE (DCL) and RNASE THREE-LIKE (RTL) endonucleases that process almost every class of double-stranded RNA precursors. Charbonnel et al. (2017) have recently demonstrated that members of DCL and RTL families in *Arabidopsis* are glutathionylated on a conserved Cys which affects their RNase III activity. R-S-SG of RTL1 is reversed by type I GRXs, suggesting that small RNA biogenesis and subsequent gene expression responses are under the control of the cell redox environment (Charbonnel et al., 2017). Indeed, the RNase activity of another member of the family (RTL2) was previously shown to be regulated by its dimerization state through an intermolecular disulfide bond (Comella et al., 2008), showing that a redox switch might regulate small RNA biogenesis.

Epigenetic regulation of gene expression is performed by DNA methylation. Some key metabolic enzymes involved in DNA methylation are suspected to be redox regulated. Among them are enzymes of the S-Adenosyl Methionine (SAM) cycle which provide precursors for DNA and histone methylation (Shen et al., 2016). Other nuclear candidates are the DNA demethylases Repressor of Silencing1 (ROS1) and Demeter-like (DME, DML2, and DML3) enzymes which remove methylated bases from the DNA backbone (Zhu, 2009). All these enzymes contain an iron-sulfur (Fe-S) cluster which might be susceptible to oxidation by ROS. Moreover, different members of the cytosolic Fe-S cluster assembly machinery (i.e., MET18 and AE7) are involved in DNA methylation, likely because they affect the nuclear DNA demethylases Fe-S cluster metabolism (Luo et al., 2012; Duan et al., 2015). Therefore, all these examples show an emerging link between redox regulation and epigenetic regulation.

## Conclusion and Perspectives

Data supporting the role of redox regulation in nuclear functions are rapidly increasing. ROS are key actors of this regulation, influencing gene expression at multiple levels (transcription and post-transcription), notably by modulating activities of transcription regulators. While H_2_O_2_ has been detected in the nucleus, little is known about ROS metabolism and dynamics in this cell compartment. The recent discovery of a H_2_O_2_ flux from the chloroplast to the nucleus opens new perspectives to decipher the role of ROS in gene expression. In this way, the identification of a redox regulation of key transcriptional regulators (e.g., transcription factors, HDAC) will shed light on the ways ROS are acting on gene expression. Key for these questions are the proteomic approaches aiming to identify nuclear PTM occurring on redox-sensitive residues, and the structure biology techniques designed to visualize redox-based modifications on protein structure (Waszczak et al., 2014; Parker et al., 2015; Zaffagnini et al., 2016; Pérez-Pérez et al., 2017). While those redox proteome approaches have identified hundreds of nuclear proteins which could be prone to redox modifications, biochemical and functional evidence is missing to support the biological significance of these redox switches. This will be a major challenge for future research in redox biology.

## Author Contributions

LM, JT-H and J-PR wrote the paper. LM and JT-H performed the figures.

## Conflict of Interest Statement

The authors declare that the research was conducted in the absence of any commercial or financial relationships that could be construed as a potential conflict of interest.
